# Radiological and biomechanical assessment of displaced greater tuberosity fractures

**DOI:** 10.1007/s00264-018-4170-x

**Published:** 2018-09-30

**Authors:** Richard W. Nyffeler, Angela Seidel, Stefan Werlen, Mathias Bergmann

**Affiliations:** 1 Orthopädie Sonnenhof, Salvisbergstrasse 4, 3006 Bern, Switzerland; 2 Department of Orthopaedic Surgery, Inselspital, University Hospital, Bern, Bern, Switzerland; 3 Klinik Sonnenhof, Bern, Switzerland; 4 Department of Anatomy, University of Bern, Bern, Switzerland

**Keywords:** Shoulder, Greater tuberosity, Fracture, Subacromial, Impingement, Index

## Abstract

**Purpose:**

Greater tuberosity fractures are challenging lesions concerning decision-making. In order to improve our treatment algorithm, we developed a new method, which allows predicting a possible subacromial conflict on standard anteroposterior radiographs, considering not only the displacement of the fragment but also the width of the subacromial space.

**Methods:**

The measurement technique consisted of drawing three concentric circles on true anteroposterior radiographs. The inner circle (radius Rh) perfectly matched the humeral head surface. The medial circle (radius Rt) was tangent to the greater tuberosity, and the outer circle (radius Ra) touched the undersurface of the acromion. The ratio Rt/Rh, which describes how much the greater tuberosity projects above the articular surface, and the relationship (Rt-Rh)/(Ra-Rh), which quantifies the space occupied by the greater tuberosity under the acromion, were calculated and called Greater Tuberosity Index and Impingement Index, respectively. Five dry humeri were used to assess the influence of rotation and abduction on the Greater Tuberosity Index. The radiographs of 80 shoulders without any osseous pathology were analyzed to obtain reference values for both indices. Finally, greater tuberosity fractures with different displacements were created in five cadaver specimens, and subacromial impingement was correlated with these parameters.

**Results:**

On anteroposterior radiographs, the greater tuberosity was most prominent in neutral rotation, regardless of abduction. In shoulders without osseous pathology, the Greater Tuberosity Index and the Impingement Index averaged 1.15 (range 1.06–1.28) and 0.46 (range 0.21–0.67). In the biomechanical experiments, the Impingement Index was a better discriminator for subacromial impingement than the Greater Tuberosity Index. A fracture with a displacement corresponding to an Impingement Index of 0.71 or greater was associated with subacromial impingement.

**Conclusions:**

Reduction of a displaced greater tuberosity fragment should be considered if the Impingement Index is 0.7 or greater. The measurement method is simple and reliable and has the potential to be used for the assessment of subacromial impingement in other conditions.

## Introduction

Fractures of the greater tuberosity are common injuries in young and elderly patients. Their incidence has been estimated to be 20% of all proximal humerus fractures [1, 2]. They can occur isolated, often during a fall or an anterior shoulder dislocation, or be part of a more complex humeral head fracture. Their treatment depends on the amount of displacement, the stability of the fragments, and the expectations of the patients. Displaced fragments may cause subacromial impingement or even hinder abduction and external rotation [3–7]. The amount of displacement that is still compatible with pain-free normal range of motion is debated, and the method to measure the displacement is not well defined in the literature [8].

Most articles concerning greater tuberosity fractures refer to the work of McLaughlin [3], Neer [4], and Park et al. [6]. In 1963, McLaughlin [3] stated that a displacement of more than 0.5 cm but less than 1 cm usually results in a convalescence in excess of six months, some permanent pain and disability, and in about 20% in a late operation for reduction of the displacement. In 1970, Neer [4] considered all humeral head fractures, regardless of the level or number of fracture lines, in which no segment was displaced more than 1.0 cm or angulated more than 45°, as minimum displaced fractures, that could be treated with a brief period of immobilization and early functional exercises. In 1997, Park et al. [6] suggested that a greater tuberosity fragment should be mobilized, repaired, and fixed into its original bed, if the displacement is more than 5 mm in young active patients and more than 3 mm in individuals involved in overhead activities or heavy labour.

**Fig. 1:**
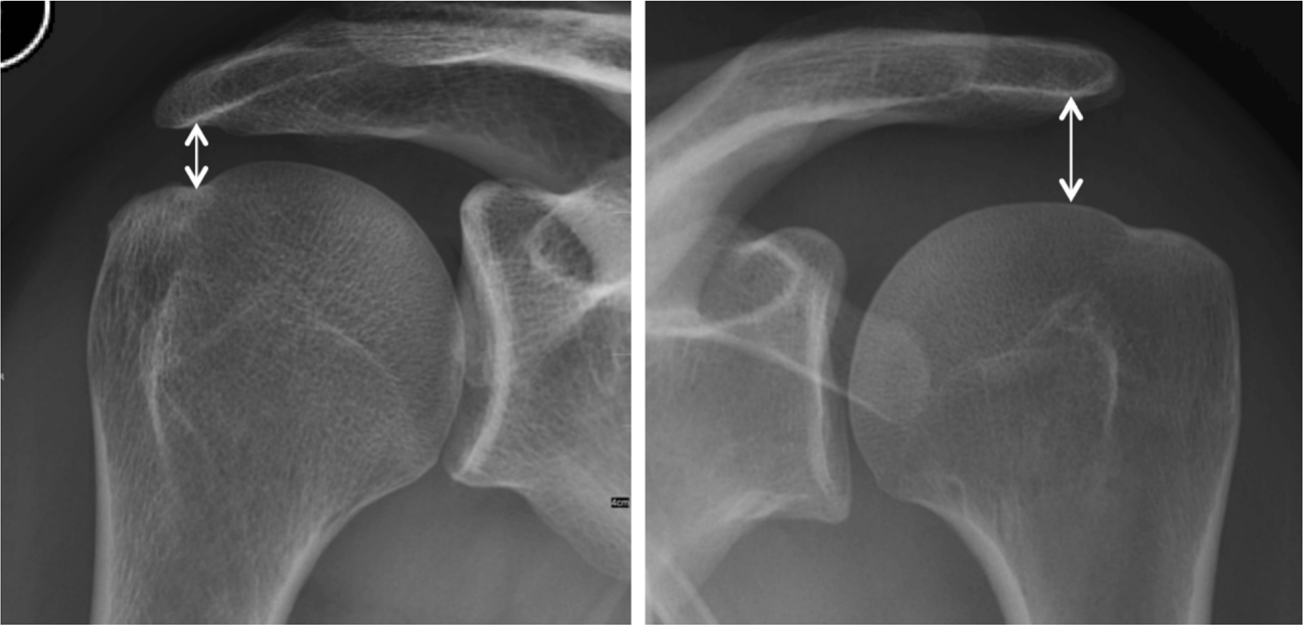
Anteroposterior radiographs of two shoulders in neutral rotation demonstrating that the acromiohumeral distance varies between individuals

None of these authors considered the anatomy of the scapula, even though the acromiohumeral distance of normal shoulders ranges from 7 to 14 mm [9–12] (Fig. 1). It can therefore be supposed that a minimally displaced greater tuberosity fragment may cause a subacromial conflict in patients with a small acromiohumeral distance and that it does not cause a problem in patients with a wider subacromial space. The purpose of the present study was therefore to develop a new method, which allows to quantify the displacement of greater tuberosity fractures on true anteroposterior radiographs and to predict a possible subacromial conflict, considering each individual’s anatomy.

## Material and methods

### Definitions and measurements

For the purpose of simplification, we assumed that the humeral head is spherical, that the thickness of the articular cartilage is constant, and that the head remains centered on the glenoid cavity in the mid-range of motion. With these assumptions, the geometric center of the articular surface corresponds to the center of rotation, and all points on the humeral head move on concentric spheres. During normal glenohumeral flexion and abduction, the greater tuberosity passes under the acromion. A subacromial conflict must be suspected, if the sphere, which is tangent to the outmost point of the greater tuberosity, touches the undersurface of the acromion. Determining the center of rotation and drawing a sphere through the outmost point of a displaced greater tuberosity fragment should therefore allow predicting a possible subacromial conflict. For simplification and because CT scans are not always available for decision-making of greater tuberosity fractures, we assessed the displacement of the fragment on two-dimensional x-ray pictures rather than three-dimensional reconstructions of CT scans.

**Fig. 2:**
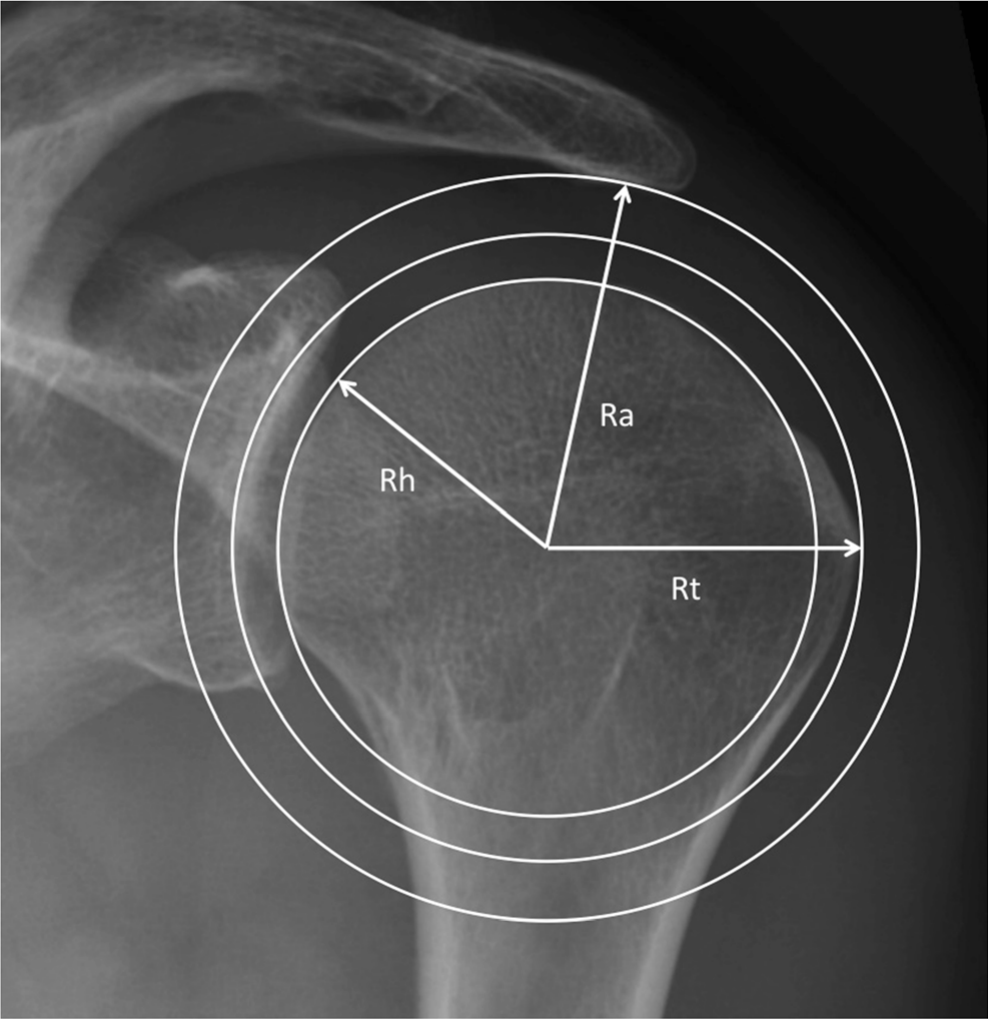
True anteroposterior radiograph of a left shoulder illustrates the measurement technique used in this study. The inner circle perfectly matches the humeral head surface (Rh) and determines the center of rotation. The middle and outer circles are concentric to the inner circle and tangent to the greater tuberosity (Rt) and the undersurface of the acromion (Ra), respectively

Three concentric circles were therefore drawn on standardized anteroposterior radiographs and the corresponding radii were measured. The inner circle (radius Rh) was drawn in such a way that it perfectly matched the subchondral bone of the humeral articular surface. Its centre corresponded to the geometric centre of the humeral head. The middle circle (radius Rt) was tangent to the greater tuberosity, and the outer circle (radius Ra) touched the undersurface of the acromion (Fig. 2).

In order to describe how much the greater tuberosity projects above the articular surface and how much room it occupies in the subacromial space, the relationships Rt/Rh (Greater Tuberosity Index) and (Rt-Rh)/(Ra-Rh) (Impingement Index) were calculated. Using relationships rather than absolute values enabled us to eliminate possible magnification errors on the x-ray images.

### Influence of rotation and elevation

**Fig. 3:**
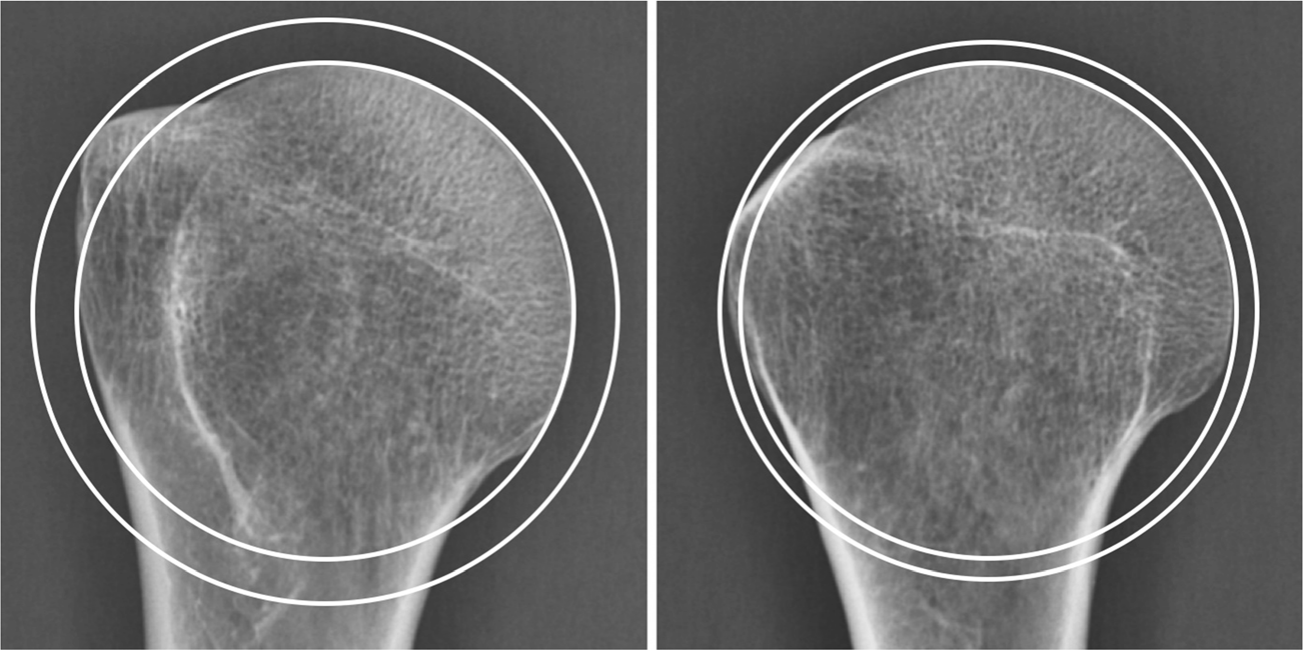
Anteroposterior radiograph of a dry humerus in neutral and internal rotation demonstrating the influence of rotation on the Greater Tuberosity Index. Internal rotation (on the right side) exposes the posteroinferior aspect and hides the superior part of the greater tuberosity, which is relevant for subacromial impingement. The measurements should therefore be made with the arm in neutral rotation

In a first step, we assessed the influence of rotation and elevation of the arm on the Greater Tuberosity Index. This was done with use of five dry humeri without osseous pathologies obtained from our Institute of Anatomy. All bones were from the right side. They were fixed one after the other on a specially designed frame, first in a vertical position and then in 30° of abduction. In both positions, the humeri were turned around their shaft axis in steps of 10° from 60° of external to 70° of internal rotation (Fig. 3). In order to compare the results with standardized radiographs taken in the clinic, neutral rotation was defined as the position, in which the epicondylar axis was 30° externally rotated relative to the X-ray cassette. In each position, a radiograph was made with the X-ray beam downwards-tilted 20° and the Greater Tuberosity Index was determined. Descriptive statistics were made using R (R Foundation for statistical computing, Vienna, Austria).

### Reference values of normal shoulders

In a second step, we searched our database and determined the Greater Tuberosity Index and the Impingement Index on 80 anteroposterior radiographs of patients treated in our clinic for different shoulder pathologies (AC joint disease *n* = 20, frozen shoulder *n* = 20, calcific tendinopathy *n* = 20, and subacromial bursitis *n* = 20). Only radiographs of adult patients without previous operations and without fractures were included. There were 36 men and 44 women and 47 right and 33 left shoulders. All pictures needed to be made with the arm in neutral rotation and the glenoid perpendicular to the x-ray cassette. The humeral head needed to be perfectly centered on the glenoid cavity. All images had been made for diagnostic purposes; no radiographs of healthy volunteers were included. In order to determine the intra- and inter-rater reliabilities, two independent observers analyzed 50 radiographs at two different times. A multivariate analysis was made using R (R Foundation for statistical computing, Vienna, Austria), and intraclass correlation coefficients were determined with the SPSS software (IBM, Armonk, USA).

### Biomechanical experiments

**Fig. 4:**
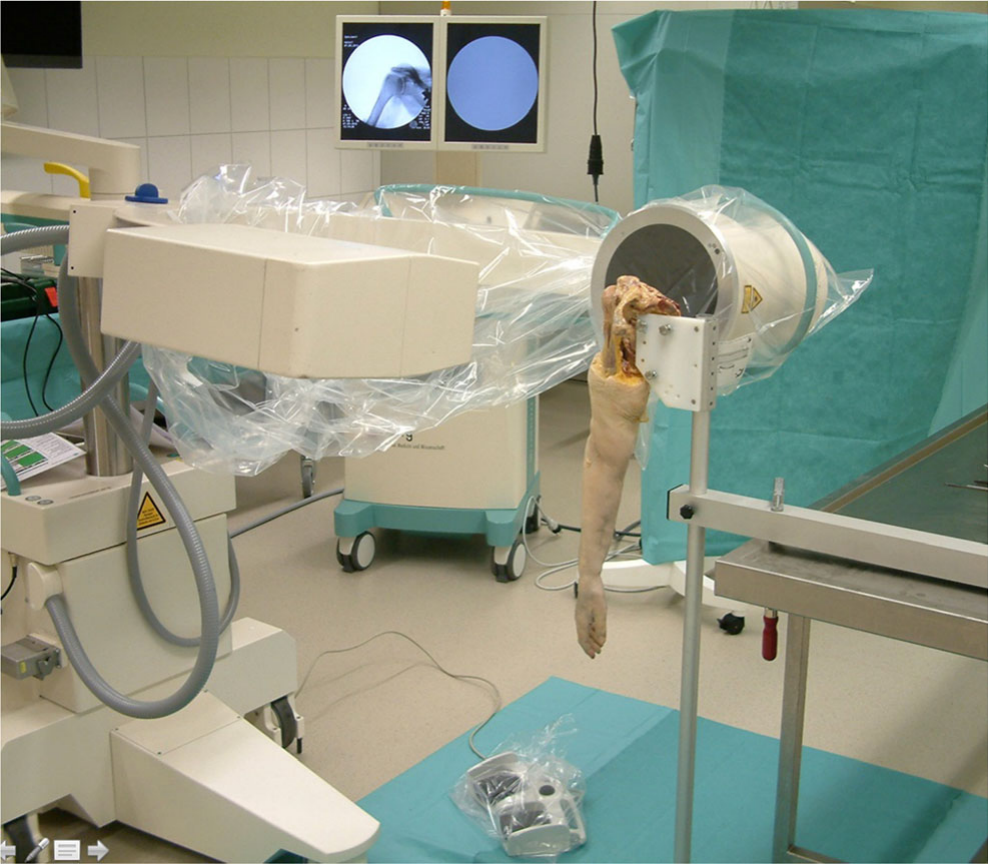
Photograph showing the experimental setup. The scapula was firmly attached to a special frame in a vertical position

**Fig. 5:**
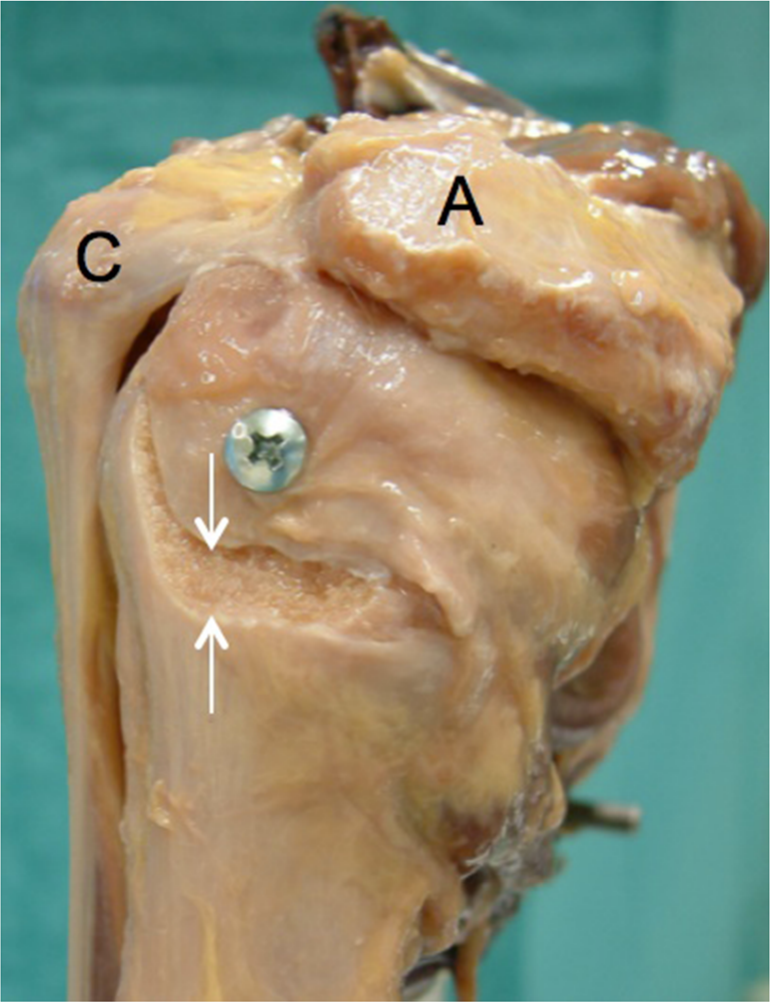
Lateral view of a left shoulder specimen with a superiorly displaced greater tuberosity fragment. The displacement was measured at the inferior tip of the fragment with a caliper. A acromion; C coracoid process

**Fig. 6:**
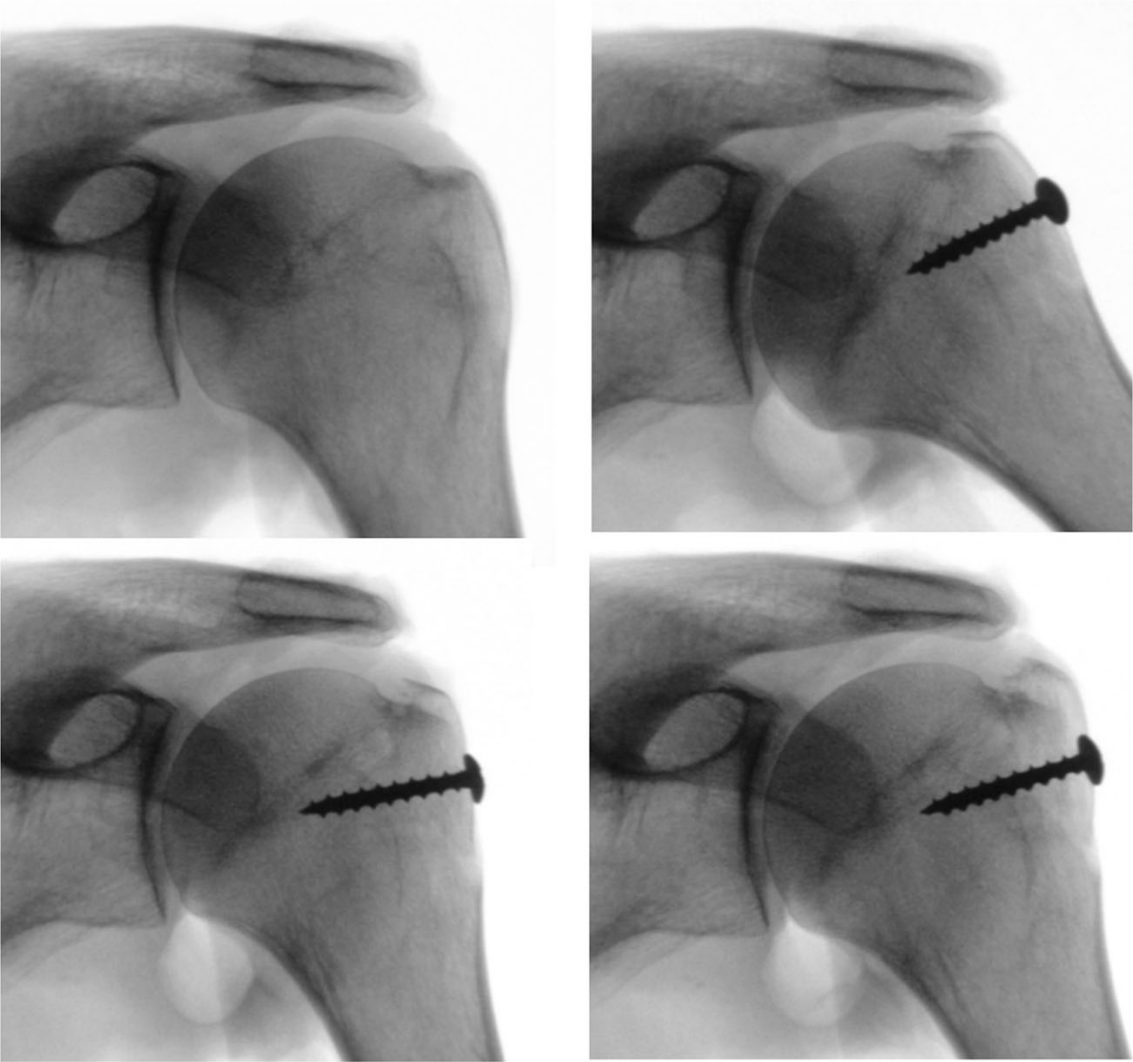
Fluoroscopy pictures of a cadaver shoulder, intact and with a greater tuberosity fragment fixed in different malpositions. The humeral head was manually centered on the glenoid

Ten Thiel-embalmed [13] upper extremities of unknown age and sex were obtained from our Institute of Anatomy and used according to the Guidelines of the Swiss Academy of Medical Sciences. Donors had formally agreed the use of body parts for research purposes by signing the donation forms. The skin and the deltoid muscle were removed; the coracoacromial ligament was carefully preserved. Inspection of the dissected shoulders showed a rotator cuff tear or osteoarthritis in five cases. These specimens had to be excluded. The scapulae of the remaining five specimens (one right, four left) were rigidly fixed one after the other on a specially designed jig, with the glenoid surface in a vertical plane (Fig. 4). The humeral head was manually pressed into the glenoid cavity, with the arm in a vertical position and in neutral rotation. A true anteroposterior X-ray image was then made under fluoroscopy (Ziehm vision, Ziehm Imaging GmbH, Nürnberg, Germany) with the X-ray beam downwards-tilted 20°. If the humeral head was not perfectly centered on the glenoid, the positioning procedure was repeated and the adequate image was stored as a jpeg file for further assessment. The arm of the specimen was then passively elevated and rotated while keeping the humeral head centered on the glenoid cavity. During these movements, particular attention was paid to the friction between the undersurface of the acromion and the bursal side of the rotator cuff. If the arm could be elevated without any resistance and without inferior subluxation of the humeral head on the glenoid, we considered that there was no impingement. If the bursal side of the rotator cuff got caught under the acromion or the coracoacromial ligament, a subacromial conflict was noted. Every movement was repeated several times, until the examiner (RWN) was sure if there was friction or not. Once the experiments were done with the intact specimen, an osteotomy of the greater tuberosity was made in order to simulate an isolated greater tuberosity fracture [14]. Care was taken to not damage the insertion of the rotator cuff. In each specimen, four different malpositions of the greater tuberosity fragment were examined. The fragment was either displaced 2 to 5 mm superiorly or 2 to 10 mm posteriorly and fixed to the humeral head with a screw. The displacement was measured at the tip of the fragment on the lateral side of the humeral head with use of a caliper (Fig. 5). For each malposition, an anteroposterior radiograph was made with the arm in neutral rotation (Fig. 6), and the impingement tests were repeated. All images were analyzed according to the method described above, and the results were correlated with the presence or absence of subacromial impingement.

## Results

### Influence of rotation and abduction on the Greater Tuberosity Index

The shape of the humeral head changed as the arm was rotated. On the x-ray pictures, the greater tuberosity was most prominent in neutral rotation and less salient in internal and external rotation (Fig. 3). Internal rotation exposed the posteroinferior aspect and no longer the highest point of the greater tuberosity, which is responsible for subacromial impingement. We therefore decided to determine the Greater Tuberosity Index and the Impingement Index in neutral rotation. In this position, the Greater Tuberosity Index of the dry humeri averaged 1.15 (range 1.11 to 1.20, SD 0.04). It did not significantly differ between 0 and 30° of abduction (*p* = 0.47).

### Greater Tuberosity Index and Impingement Index of shoulders without osseous pathology

**Fig. 7:**
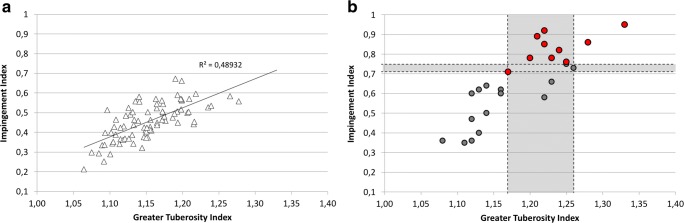
**a** Graph showing the relationship between Greater Tuberosity Index and Impingement Index of the 80 patients without osseous pathology. There was a positive but only moderate correlation between these two values (coefficient of determination *R*^2^ = 0.4893). **b** Graph showing the relationship between Greater Tuberosity Index and Impingement Index of the five cadaver specimens. Each specimen was tested intact and with a greater tuberosity fragment fixed in four malpositions. A gray dot means that no impingement could be observed, and a red dot indicates that a subacromial conflict was detected. The gray area contains pairs of values that were (red dots) or were not (gray dots) associated with subacromial impingement. The width of these bands indicates that the Impingement Index was a better discriminator for subacromial impingement than the Greater Tuberosity Index

The measurement technique proved to be reproducible. The intra- and inter-observer agreements were good to excellent [15] with intraclass correlation coefficients of 0.89 and 0.88 for the Greater Tuberosity Index and 0.89 and 0.85 for the Impingement Index. The results of the radiographic study are reported in Fig. 7a and Table 1. There was a significant positive but only moderate correlation between the Greater Tuberosity Index and the Impingement Index (Pearson correlation coefficient *R* = 0.699). Female patients had significantly higher values than male patients, for both the Greater Tuberosity Index (1.17 vs 1.13; *p* = 0.0002) and the Impingement Index (0.48 vs 0.46; *p* = 0.02). There was a non-significant positive correlation between the Greater Tuberosity Index and age (Pearson correlation coefficient 0.183) and a significant positive but poor correlation between the Impingement Index and age (0.255). Neither the diagnosis nor the side had a significant influence on these indices.

**Table 1:** The diagnosis, the age, and the radiographic parameters of the 80 patients included in the study. The differences between pathologies were not significant

Diagnosis	Age	Greater Tuberosity Index	Impingement Index
	avg (SD; range)	avg (SD; range)	avg (SD; range)
AC joint disease (*n* = 20)	45.4 (13; 18–69)	1.13 (0.03; 1.08–1.20)	0.43 (0.09; 0.25–0.58)
Frozen shoulder (*n* = 20)	53.1 (11; 27–78)	1.15 (0.04; 1.09–1.21)	0.46 (0.08; 0.34–0.57)
Calcific tendinopathy (*n* = 20)	54.1 (11; 39–76)	1.17 (0.04; 1.09–1.28)	0.45 (0.08; 0.32–0.67)
Subacromial bursitis (*n* = 20)	42.4 (13; 18–57)	1.17 (0.05; 1.06–1.27)	0.48 (0.12; 0.21–0.66)
All (*n* = 80)	48.8 (13; 18–78)	1.15 (0.04; 1.06–1.28)	0.46 (0.09; 0.21–0.67)

### Relationship between displacement of the greater tuberosity fragment, Greater Tuberosity Index, Impingement Index, and subacromial impingement

The Greater Tuberosity Index and the Impingement Index of the five cadaver shoulders averaged 1.16 (SD 0.07; range 1.08 to 1.23) and 0.51 (SD 0.15; range 0.35 to 0.66), respectively. In none of the intact specimens, a subacromial impingement could be detected. A superior displacement of the greater tuberosity fragment of 3 mm resulted in a subacromial impingement in all specimens. A posterior displacement of 5 mm caused a subacromial impingement in half of the specimens, whereas a posterior displacement of 10 mm resulted in an impingement during abduction with internal rotation in all cases. The relationship between Impingement Index and Greater Tuberosity Index is shown in Fig. 7b. If the Impingement Index was less than 0.71 and/or if the Greater Tuberosity Index was less than 1.17, no impingement could be detected. A displacement of the fragment corresponding to an Impingement Index of more than 0.75 and/or a Greater Tuberosity Index of more than 1.26 was always associated with subacromial impingement. Between these values, subacromial impingement could be present or not. The subacromial conflict occurred at very low abduction angles, typically at 20 to 30° of glenohumeral abduction.

## Discussion

Current recommendations for the treatment of greater tuberosity fractures are based on a small number of clinical and radiographic studies made with few patients. Conservative treatment is recommended, if the displacement is less than 5 mm in the general population [3, 16–18] or less than 3 mm in overhead athletes and heavy labourers with overhead activities [6]. In our biomechanical study, a superior displacement of the greater tuberosity of 3 mm resulted in a subacromial impingement in all cases. This is not surprising since a lot of patients suffer from subacromial impingement without a previous fracture of the greater tuberosity.

Instead of measuring the displacement of the supraspinatus footprint at the fracture site, we quantified the displacement of the supero-lateral aspect of the greater tuberosity, which is more relevant for subacromial impingement. Our method also considers the width of the subacromial space, which differs between individuals and which is crucial for the presence or absence of impingement. The correlation between Impingement Index and Greater Tuberosity Index of normal shoulders was only moderate. This confirms that subacromial impingement cannot be predicted reliably when considering the position of the greater tuberosity alone.

Two radiographs taken with the arm in neutral rotation (ap- and lateral) and a medical image viewer are most often enough for decision-making. Radiographs can be obtained in all emergency departments and in most private practices, during the first consultation after the injury and during follow-up controls. The anteroposterior view should be of good quality, with the glenoid orthogonal to the x-ray cassette, the arm held in neutral rotation, and the humeral head centered on the glenoid cavity. The undersurface of the acromion should be well defined. Many patients arriving at the emergency department hold their arm against the upper body to avoid pain. Internal rotation exposes the posterior aspect of the greater tuberosity and hides the supraspinatus footprint on ap-views. Decision-making is much easier on standardized images. The patient or the physician should therefore carefully turn the injured arm into neutral rotation. This is normally well tolerated after administration of a painkiller, even in the presence of a displaced three-part fracture. When a shoulder dislocation is suspected, the lateral radiograph may be done first. If the undersurface of the acromion is not well defined, it may be more difficult to draw the accurate acromion circle. In such a case, we propose to change the inclination of the x-ray beam and to take another anteroposterior radiograph. The undersurface of the acromion should be visible as sclerotic line. If the humeral head is not perfectly centered on the glenoid, for instance because of a concomitant lesion of the axillary nerve, then the displacement of the greater tuberosity fragment cannot be determined with the Impingement Index. In these cases, the Greater Tuberosity Index may be used for decision-making. However, its critical value is less well defined than that for the Impingement Index (Fig. 7b).

**Fig. 8:**
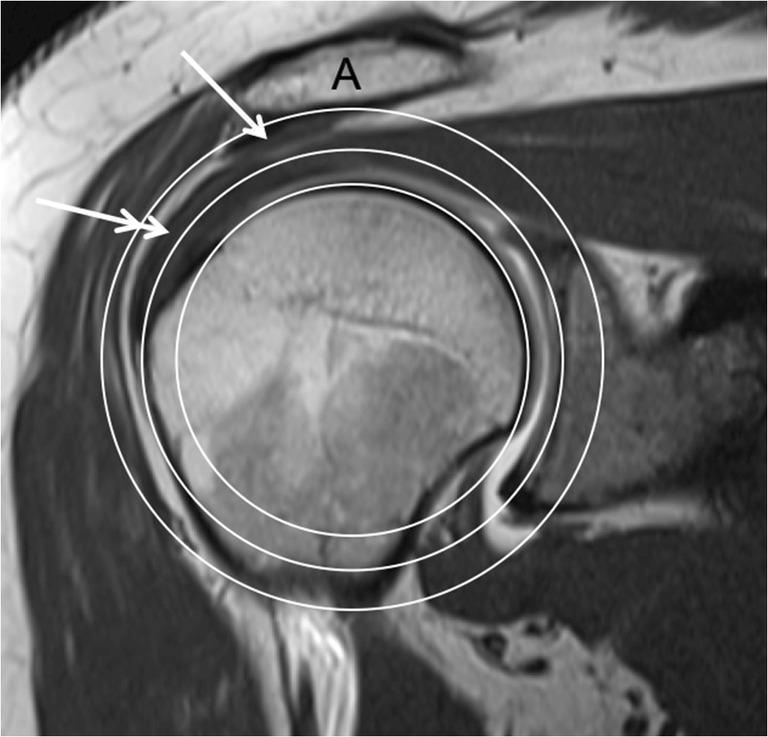
MRI of a right shoulder demonstrating that the supraspinatus tendon projects above the circle (double arrow), which is tangent to the greater tuberosity, and that the strong coracoacromial ligament (arrow) is inside the circle, which touches the undersurface of the acromion. This explains why a subacromial impingement could be observed in the cadaver experiments for an Impingement Index inferior to 1.0

In the cadaver experiments, an Impingement Index of 0.71 and higher was associated with subacromial impingement in most cases. The fact that impingement occurred at a value below 1.0 means that the conflict was not caused by a bony contact of the greater tuberosity with the acromion. This can be illustrated on a coronal MR image on which the three circles are drawn (Fig. 8). The bursal side of the supraspinatus tendon is further away from the center of rotation than the outmost point of the greater tuberosity. Additionally, the coracoacromial ligament attached on the undersurface of the acromion decreases the subacromial space. It is therefore logical that the supraspinatus tendon rather than the greater tuberosity impinges against the undersurface of the acromion or the coracoacromial ligament. This pathomechanism is consistent with the literature concerning impingement syndrome [19–21]. CT scans with three-dimensional reconstruction of the shoulder and animation of the bones would therefore not correctly simulate subacromial impingement.

The above-described measurement method was developed for the evaluation of greater tuberosity fractures. Given its simplicity and reliability, it can be used for the study of subacromial impingement without a fracture [22] and for the quality control after humeral head replacement [23] and osteosynthesis as well.

This study has some limitations. The number of shoulder specimens that could be tested is small. But it corresponds to the number of specimens used in each subgroup of a recently published biomechanical study [24]. We created only a simple fracture of the greater tuberosity, although there may be many different fracture patterns in reality. However, the relevant criterion for subacromial impingement is not the number of fragments but the position of the outmost piece of bone relative to the centre of rotation. We decided to use a single fragment with a reasonable size because it could be fixed to the cancellous bone more securely. This fragment was displaced superiorly or posteriorly, but not in both directions at the same time. One can assume that posterosuperior displacement is more relevant than posterior displacement alone, but less critical for subacromial impingement than superior displacement [25]. Since the x-ray beam of standard anteroposterior radiographs is downwards tilted, relevant posterosuperior displacements can easily be recognized and analyzed with our technique. The method is based on the assumption that the geometric centre of the bony surface of the humeral head corresponds to the centre of rotation of the glenohumeral joint and that the head remains centered during active motion. It is possible that the instantaneous centre of rotation of the humeral head is slightly different and that activation of the shoulder muscles in low abduction angles results in a small upward translation of the humeral head on the glenoid. One could therefore assume that the experimentally determined critical value of 0.7 for the Impingement Index could be smaller in vivo. Additional clinical and radiographic studies with patients having a greater tuberosity fracture could confirm or correct this experimentally determined value. Previous biomechanical studies investigating subacromial impingement and greater tuberosity fractures used pressure-sensitive films [26] or a dynamic shoulder testing apparatus [7]. Any device placed under the acromion decreases the subacromial space and therefore falsifies the results. A shoulder simulator with some actuators may be adequate for coarse movements and force measurements in a single plane, but it cannot reproduce more complex movements such as flexion or abduction combined with internal rotation. It is also not sensitive enough to detect subtle friction under the coracoacromial arc. We therefore performed our experiments manually and carefully observed what happened during passive shoulder motion in all directions. Despite all the limitations, we are convinced that our measurement method using concentric circles is reliable and useful for further research and clinical application.

## Conclusions

This study presents a simple and reliable method to quantify the displacement of greater tuberosity fractures and predict a subacromial impingement on standard anteroposterior radiographs. The results suggest that a displaced greater tuberosity fragment may cause a subacromial conflict if the Impingement Index is equal or greater than 0.7. The method has the potential to be used for the assessment of subacromial impingement in patients without a fracture and in patients who had an osteosynthesis of the humeral head or a shoulder replacement.
